# Supplementary material for: Temporal asymmetries in inferring unobserved past and future events
Source: Nat Commun. 2024 Oct 1;15:8502. doi: 10.1038/s41467-024-52627-5 (PMC11445511; doi:10.1038/s41467-024-52627-5)
Supplement: Supplementary file 1 — Supplementary Information [file 41467_2024_52627_MOESM1_ESM.pdf]

Supplementary materials for: Temporal asymmetries in inferring unobserved past and future events

Xinming Xu[1], Ziyan Zhu[2], Xueyao Zheng[3], and Jeremy R. Manning[1,⋆]

[1]Dartmouth College, Hanover, NH, USA

[2]Peking University, Beijing, China

[3]Beijing Normal University, Beijing, China

[⋆]Address correspondence to jeremy.r.manning@dartmouth.edu

September 12, 2024

Table S1: **Stimulus descriptions (main experiment).**

Storyline	Segment	Episode	Duration (s)	Main characters	Number of events[a]	
					Onscreen	Offscreen[b]
1	1	1	105	Beth, Sheila, Rob, Leo	4	7
1	2	1	172	Beth, Sheila, Rob, Leo	7 [1]	3
1	3	1	70	Beth, Sheila, One neighbor	6	2
1	4	1	135	Beth, Sheila	8 [1]	1
1	5	1	90	Beth, Rob, April	8	4
1	6	1	188	Beth, Rob	6 [2]	1
1	7	1	91	Beth, Sheila	5 [1]	2
1	8	1	142	Beth, April	4 (1) [2]	3 (1)
1	9	2	134	Beth, April	4 [2]	1
1	10	2	58	Beth	3 [1]	6 [1]
1	11	2	159	Beth, Rob	9	
2	1	1	70	Simone, Karl	3 [1]	6
2	2	1	119	Simone, Karl, Naomi, Tommy	4 [1]	2
2	3	1	58	Simone, Karl, Tommy	4	2
2	4	1	102	Simone, Karl	4	6
2	5	1	134	Simone, Karl, Tommy	6 (3) [1]	3
2	6	1	81	Simone, Karl, Neighbors, Wanda	4 (1)	6
2	7	1	194	Simone, Tommy	8	3
2	8	2	101	Simone, Karl	3 (1)	1
2	9	2	86	Simone, Tommy	4 [1]	3
2	10	2	232	Simone, Karl	6 [1]	3
2	11	2	189	Simone, Naomi, Tommy	7 (1) [1]	

[a] Number of partial events (see methods) shown in parentheses; number of summary events shown in square brackets.
[b] Offscreen events happened between the current segment and the next segment.

Table S2: **Stimulus descriptions (replication experiment).**

Segment	Duration (s)	Main characters	Number of events[a]	
			Onscreen	Offscreen[b]
1	258	Ji-Yoon, All faculty; Bill, Doodles[c]	14 (1) [1]	1
2	40	Joan, All faculty	3 (2)	0
3	87	Dean, Ji-Yoon	7 [1]	3
4	78	Elliot, Yasmine	5 [1]	1
5	66	Ji-Yoon, Yasmine	2 [1]	9 (1)
6	212	Bill; Bill, Dafna; Elliot, Yasmine	11 (2)	1
7	134	Ji-Yoon, Joan	5 (3)	0
8	38	Bill, Lila	2	0
9	35	Elliot, Yasmine	1	2 (1)
10	164	Bill, Ji-Yoon	8 [2]	5
11	154	Ji-Yoon, Habi, JuJu	5 [1]	9 [1]
12	216	Joan; Ji-Yoon, Lila; Ji-Yoon, Bill	6 [1]	0
13	56	Ji-Yoon, Bill	2 [1]	

[a] Number of partial events (see methods) shown in parentheses; number of summary events shown in square brackets.
[b] Offscreen events happened between the current segment and the next segment.
[c] Characters from different scenes are separated by ";".

Table S3: List of datasets analyzed in our meta-analysis (see Fig. 8).

Dataset	Short name	Description	Category	Number of observations	Observation type	Number of words
Internet Movie Script Database	IMSDb	A collection of transcripts from roughly 1000 popular movies.	Film	1091	Transcript	26023348
Movie Dialogues Dataset	Movies	A large collection of fictional conversations extracted from raw movie scripts.	Film	304713	Utterance	3209921
Switchboard Dialog Act Corpus	Switchboard	A collection of five-minute telephone conversations between two participants, annotated with speech act tags.	Speech	122646	Utterance	2052779
Supreme Court Corpus	SCOTUS	A collection of cases from the U.S. Supreme Court, along with transcripts of oral arguments.	Speech	1700789	Utterance	71889094
Tennis Interviews	Tennis	Transcripts for tennis singles post-match press conferences for major tournaments between 2007 to 2015.	Speech	163948	Utterance	7043118
Persuasion for Good Corpus	PfG	A collection of online conversations generated by Amazon Mechanical Turk workers, where one participant (the persuader) tries to convince the other (the persuadee) to donate to a charity.	Speech	20932	Utterance	351759
Intelligence Squared Debates Corpus	IQ2	This dataset contains transcripts of debates held as part of Intelligence Squared Debates.	Speech	26562	Utterance	1898509
Group Affect and Performance Corpus	GAP	Group members completed a Winter Survival Task (WST), a group decision-making exercise where participants must rank 15 items according to their importance in a hypothetical plane crash scenario. Participants first rank the items individually. Then, each group was given a maximum of 15 minutes to complete the WST. The group's conversations and deliberations during this task were recorded as conversations in this dataset.	Speech	8009	Utterance	45989
The Chair	Chair	Scraped transcripts from The Chair, Season 1.	Television	6	Transcript	19197
Friends Corpus	Friends	A collection of all the conversations that occurred over 10 seasons of Friends, a popular American TV sitcom that ran in the 1990s.	Television	67373	Utterance	622894
Gutenberg Dialogue Dataset	Gutenberg	Dialogues extracted from the Project Gutenberg collection.	Text	14773741	Utterance	327519461
Reddit Corpus	Reddit	A collection of Corpuses of Reddit data built from Pushshift.io Reddit Corpus. Each Corpus contains posts and comments from an individual subreddit from its inception until Oct 2018.	Text	74468	Utterance	3080662

Table S4: Part of speech pattern templates used to identify verb tenses in our meta analysis. Part of speech tags are represented between angled brackets, and pipes (|) denote instances where *any* of the two or more parts of speech are considered a match when they appear at the given position in the indicated sequence. The plus signs (+) denote that one or more repetitions of the given sequence within a single sentence are still counted as a "single" instance of the given tense. Part of speech tags are defined in Table S5.

<table>
<tr><th>Tense</th><th>Pattern template</th></tr>
<tr><td>conditional continuous</td><td><MD><BE><VBG|HVG|BEG>+</td></tr>
<tr><td>conditional continuous passive</td><td><MD><BE><BEG><VBN|VBD>+</td></tr>
<tr><td>conditional indefinite</td><td><MD><BE|DO|VB|HV>+</td></tr>
<tr><td>conditional indefinite passive</td><td><MD><BE><VBN|VBD>+</td></tr>
<tr><td>conditional perfect</td><td><MD><HV><HVN|BEN|VBN|VBD>+</td></tr>
<tr><td>conditional perfect continuous</td><td><MD><HV><BEN><VBG|HVG|BEG>+</td></tr>
<tr><td>conditional perfect passive</td><td><MD><HV><BEN><VBN|VBD>+</td></tr>
<tr><td>future continuous</td><td><MDF><BE><VBG|HVG|BEG>+</td></tr>
<tr><td>future continuous passive</td><td><MDF><BE><BEG><VBN|VBD>+</td></tr>
<tr><td>future indefinite</td><td><MDF><BE|DO|VB|HV>+</td></tr>
<tr><td>future indefinite passive</td><td><MDF><BE><VBN|VBD>+</td></tr>
<tr><td>future perfect</td><td><MDF><HV><HVN|BEN|VBN|VBD>+</td></tr>
<tr><td>future perfect continuous</td><td><MDF><HV><BEN><VBG|HVG|BEG>+</td></tr>
<tr><td>future perfect continuous passive</td><td><MDF><HV><BEN><BEG><VBN|VBD>+</td></tr>
<tr><td>future perfect passive</td><td><MDF><HV><BEN><VBN|VBD>+</td></tr>
<tr><td>infinitive</td><td><TO><BE|HV|VB>+</td></tr>
<tr><td>past continuous</td><td><BED|BEDZ><VBG|HVG|BEG>+</td></tr>
<tr><td>past continuous passive</td><td><BED|BEDZ><BEG><VBN|VBD>+</td></tr>
<tr><td>past indefinite</td><td><DOD><VB|HV|DO>|<BEDZ|BED|HVD|VBN|VBD>+</td></tr>
<tr><td>past indefinite passive</td><td><BED|BEDZ><VBN|VBD>+</td></tr>
<tr><td>past perfect</td><td><HVD><BEN|VBN|HVD|HVN>+</td></tr>
<tr><td>past perfect continuous</td><td><HVD><BEN><HVG|BEG|VBG>+</td></tr>
<tr><td>past perfect continuous passive</td><td><HVD><BEN><BEG><VBN|VBD>+</td></tr>
<tr><td>past perfect passive</td><td><HVD><BEN><VBN|VBD>+</td></tr>
<tr><td>present continuous</td><td><BEM|BER|BEZ><BEG|VBG|HVG>+</td></tr>
<tr><td>present continuous passive</td><td><BEM|BER|BEZ><BEG><VBN|VBD>+</td></tr>
<tr><td>present indefinite</td><td><DO|DOZ><DO|HV|VB>+|<DO|HV|VB|BEZ|DOZ|BER|HVZ|BEM|VBZ>+</td></tr>
<tr><td>present indefinite passive</td><td><BEM|BER|BEZ><VBN|VBD>+</td></tr>
<tr><td>present perfect</td><td><HV|HVZ><BEN|HVD|VBN|VBD>+</td></tr>
<tr><td>present perfect continuous</td><td><HV|HVZ><BEN><VBG|BEG|HVG>+</td></tr>
<tr><td>present perfect continuous passive</td><td><HV|HVZ><BEN><BEG><VBN|VBD>+</td></tr>
<tr><td>present perfect passive</td><td><HV|HVZ><BEN><VBN|VBD>+</td></tr>
</table>

Table S5: Part of speech tag definitions.

**Tag**	**Part of speech**
BE	Be
BEG	Related to beginning or gerundive form
BEM	Am (first person singular present of BE)
BEN	Been (past participle of BE)
BER	Are (second person singular and all plural present of BE)
BEZ	Is (third person singular present of BE)
BED	Were (past plural of BE)
BEDZ	Was (past singular of BE)
DO	Do
DOD	Did (past tense of DO)
DOZ	Do, 3rd person singular present
HV	Have (archaic form for historical texts)
HVD	Have, past tense (archaic)
HVG	Have, gerund or present participle (archaic)
HVN	Have, past participle (archaic)
HVZ	Have, 3rd person singular present (archaic)
MD	Modal
MDF	Modal, future tense
TO	To
VB	Verb, base form
VBD	Verb, past tense
VBG	Verb, gerund or present participle
VBN	Verb, past participle
VBP	Verb, non-3rd person singular present
VBZ	Verb, 3rd person singular present

Table S6: Keywords and phrases used to identify references to the past.

ago	elapsed	last month	olden days	thus far
already	expired	last night	once	to date
antiquity	final	last quarter	once upon a time	up to now
back when	formerly	last season	previously	used to
before	had	last semester	recently	used to be
bygone	heretofore	last time	said	was
ceased	historically	last week	since	were
concluded	hitherto	last year	so far	wrote
did	in the past	long ago	terminated	yesterday
earlier	in those days	made	then	yesteryear

Table S7: Keywords and phrases used to identify references to the future.

after	going to	later	next time	shortly
anticipated	hereafter	later on	next week	some day
can	imminently	looming	next year	soon
could	impending	may	on the horizon	subsequent
down the line	in the cards	might	plan to	succeeding
eventual	in the future	next month	predicted	to be
eventually	in the works	next quarter	prospective	tomorrow
forthcoming	in time	next season	scheduled to	upcoming
futuristic	intend to	next semester	shall	will

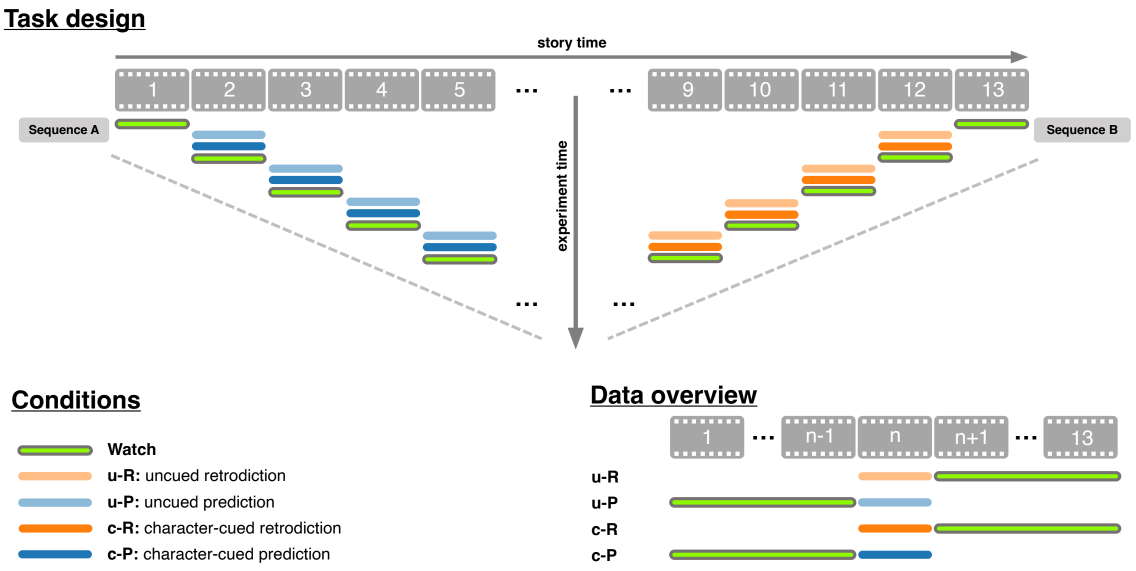

Figure S1: **Task overview (replication experiment).** Participants in our replication experiment watched segments from the television series *The Chair*. They made free-form text responses to either retrodict what had happened in the previous segment, or predict what would happen in the next segment. We systematically varied whether participants watched the segments in forward or reverse chronological order, whether (or not) responses were cued using the main characters in the target segment, and which other segments participants had watched prior to making a response. For each segment, we collected several retrodiction or prediction across different experimental conditions. Experiment time is denoted along the vertical axis, storyline segments orders are indicated along the horizontal axis, and the colors denote experimental tasks (conditions).

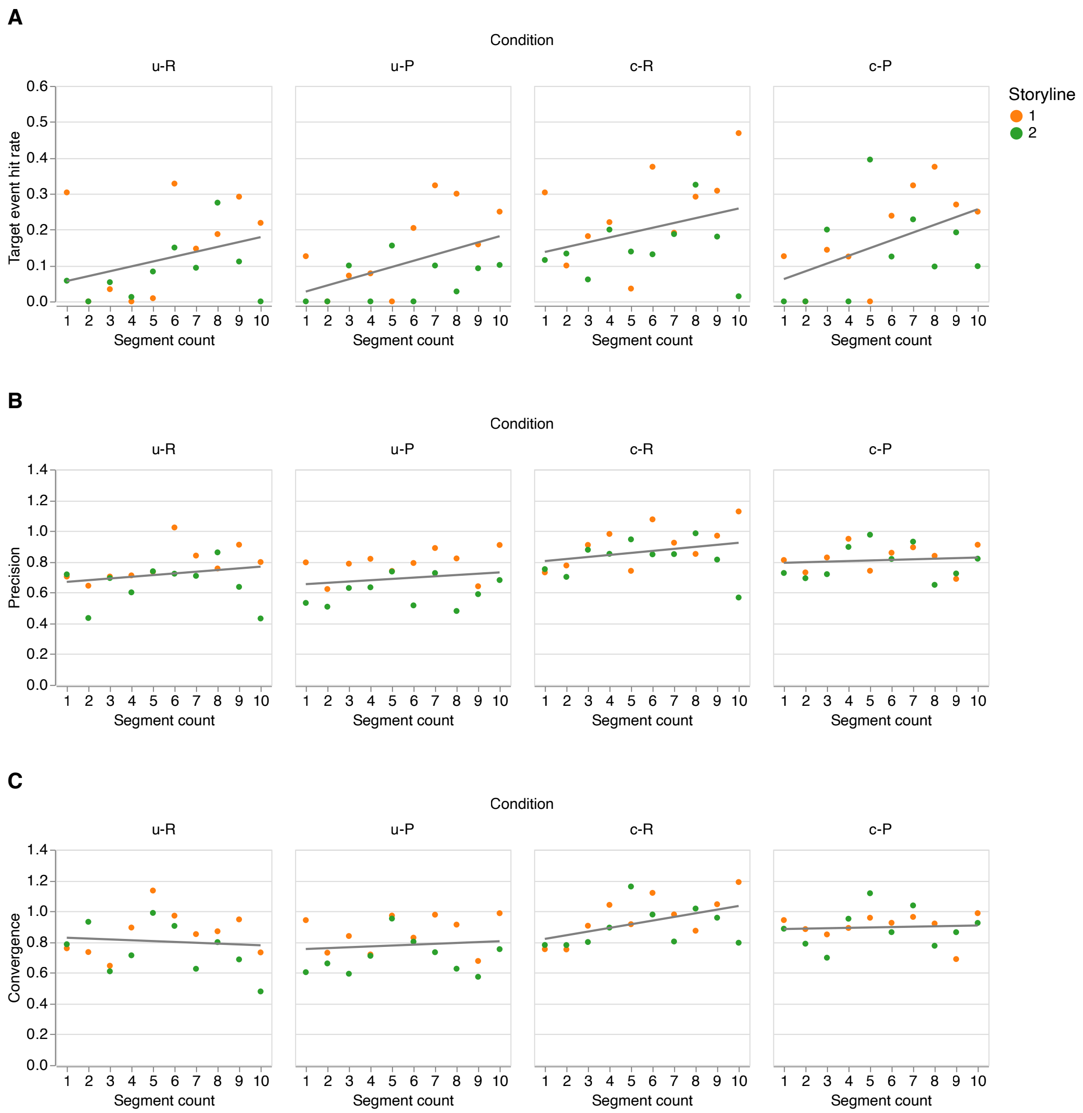

Figure S2: **Mean proportion of target events hit (A), precision (B), and convergence (C) as a function of number of segments watched in each storyline, in participants' (n=36) uncued and character-cued retrodictions and predictions (main experiment).** Grey lines represent the least squares fits.

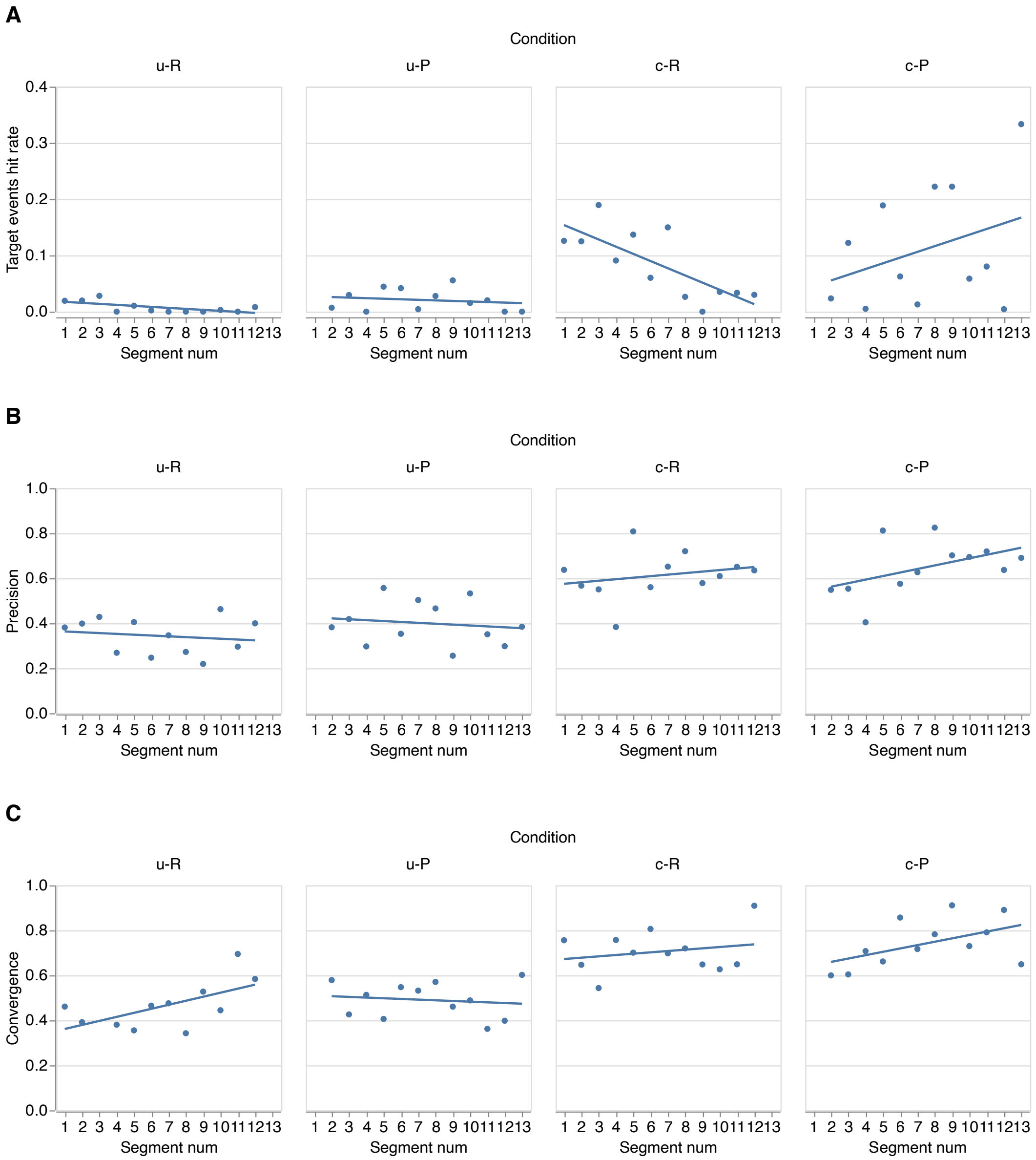

Figure S3: **Mean proportion of target events hit (A), precision (B), and convergence (C) as a function of number of segments watched, in participants' (n=37) uncued and character-cued retrodictions and predictions (replication experiment).** Blue lines represent the least squares fits.

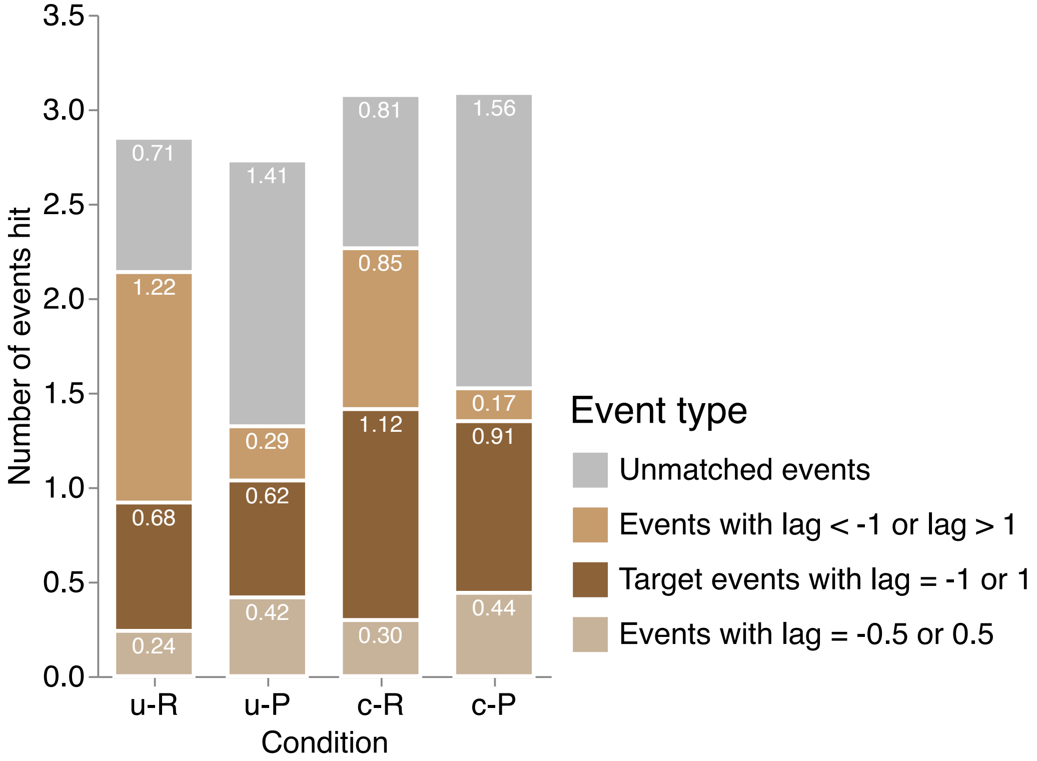

Figure S4: **Mean number of events hit for each event type, in participants' (n=36) uncued and character-cued retrodictions and predictions, averaged across just-watched segments (main experiment).**

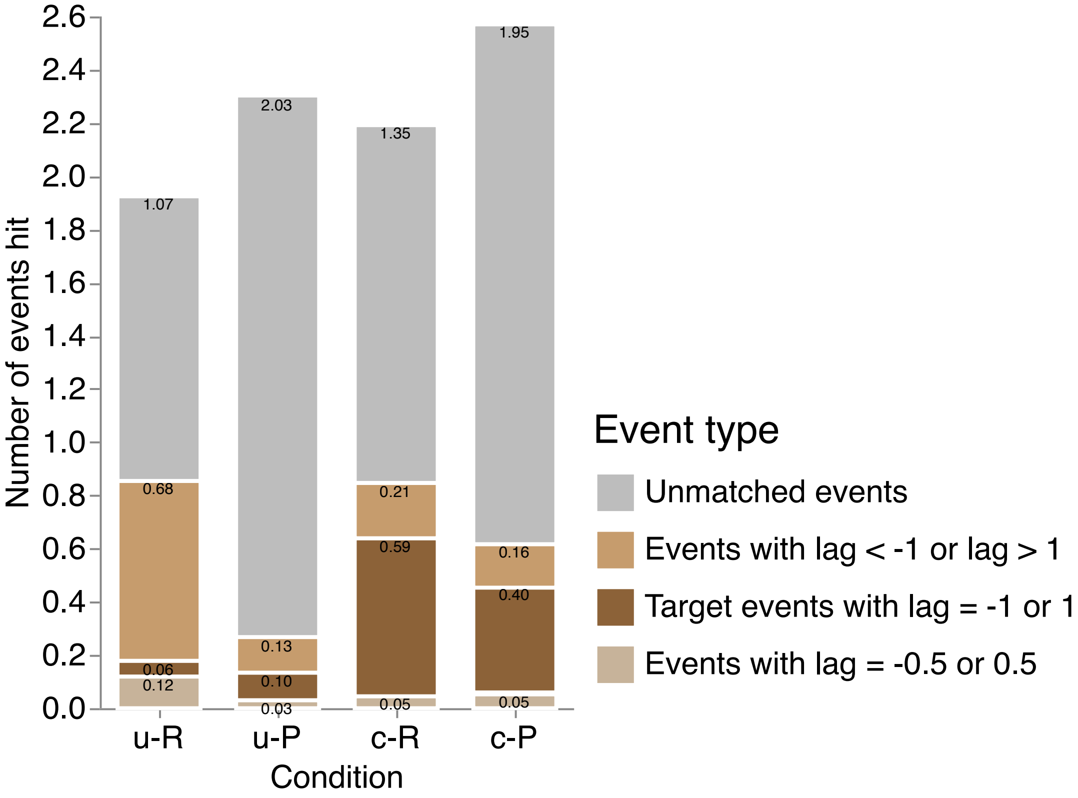

Figure S5: **Mean number of events hit for each event type, in participants' (n=37) uncued and character-cued retrodictions and predictions, averaged across just-watched segments (replication experiment).**

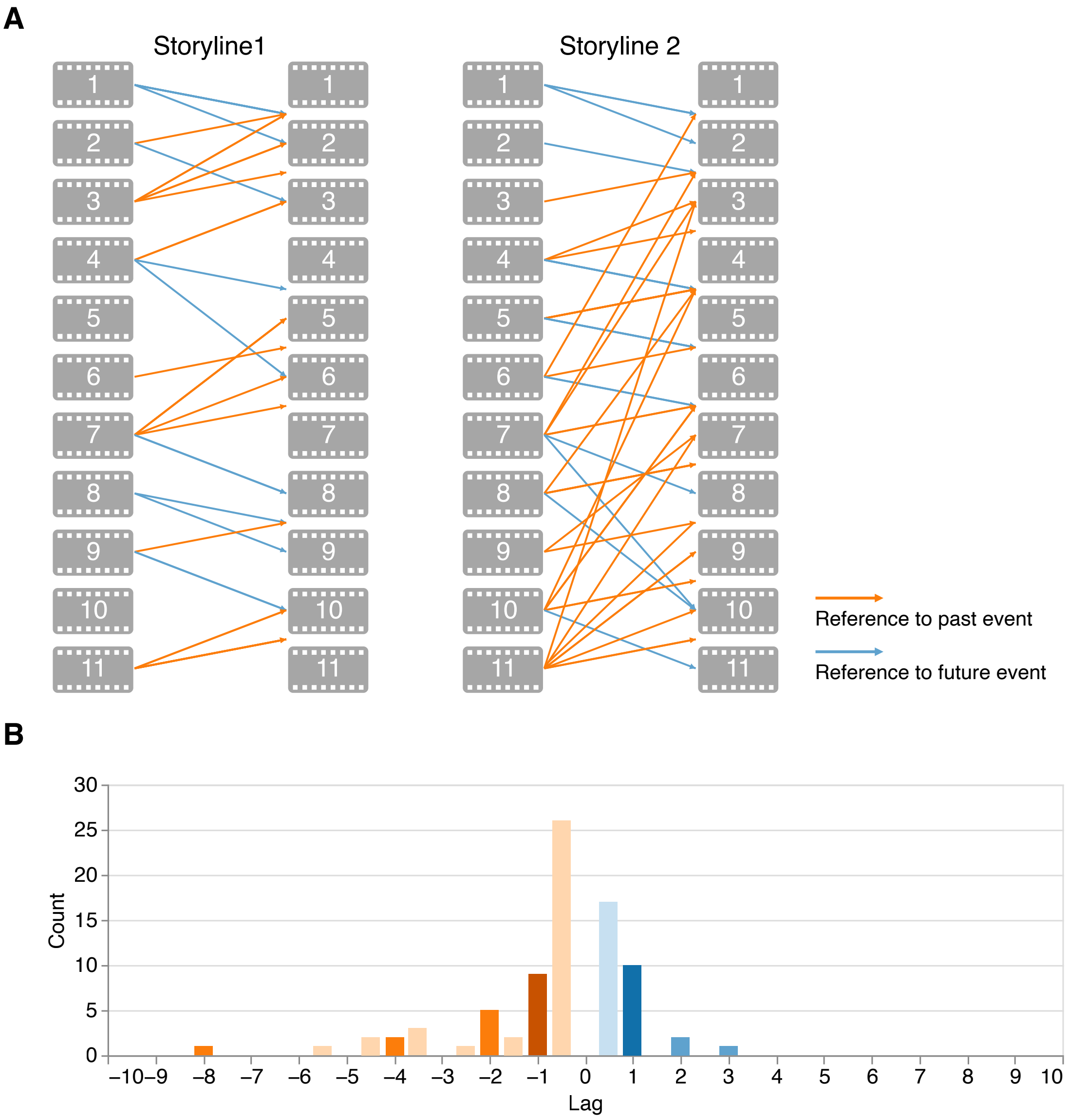

Figure S6: **(A)** All references in each storyline (main experiment). **(B)** Distribution of reference lags (main experiment).

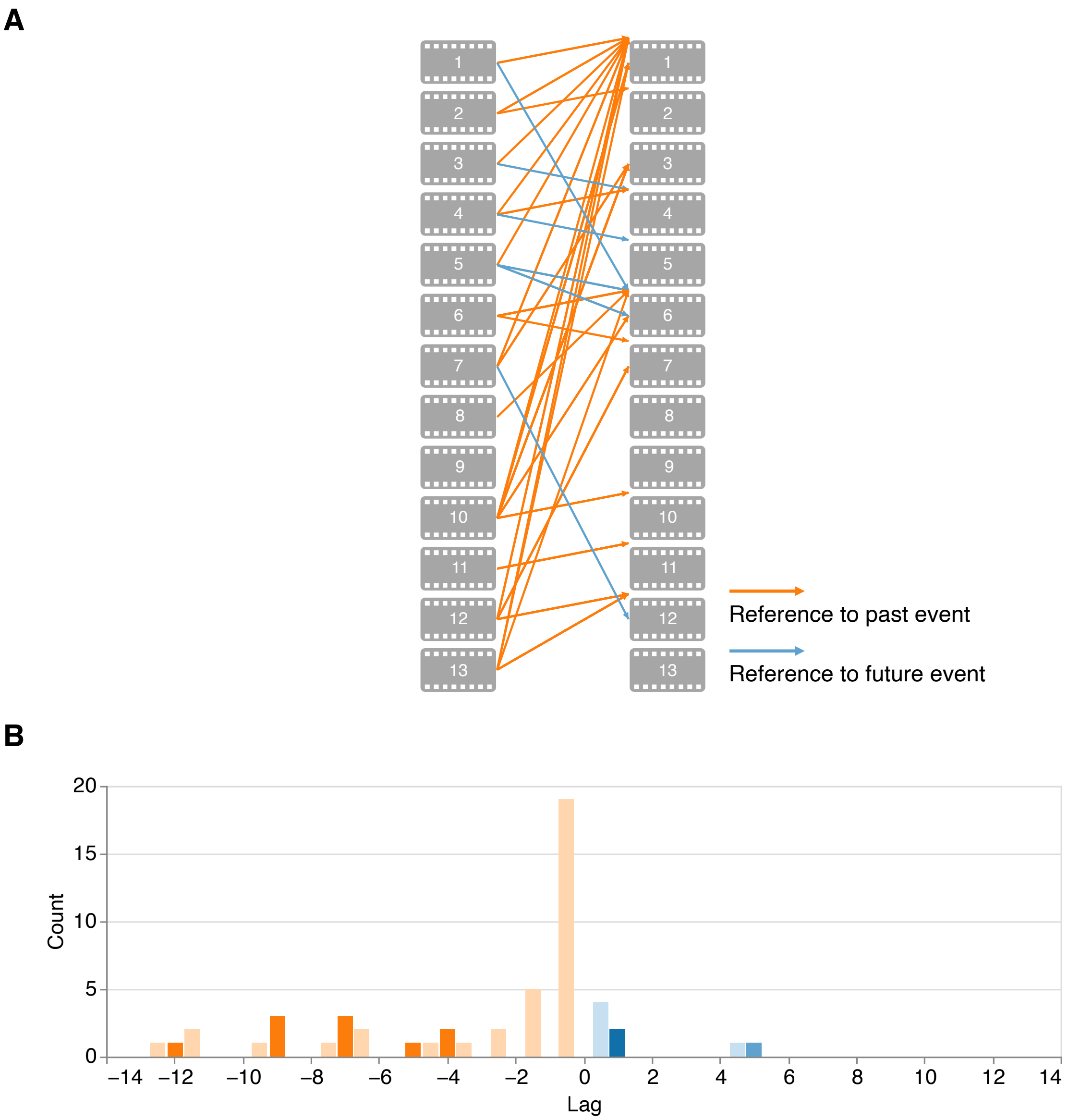

Figure S7: **(A)** All references (replication experiment). **(B)** Distribution of reference lags (replication experiment).

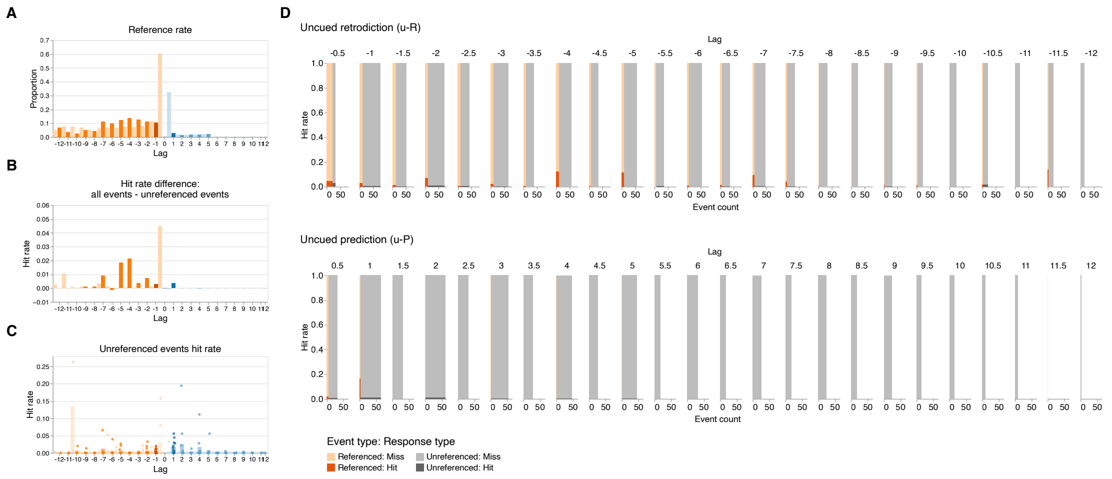

Figure S8: **Characters' references drive participants' retrodiction performance (replication experiment). A. Reference rate as a function of lag.** Across all possible just-watched segments (lag 0), the bar heights denote the average proportions of events referenced in other past or future segments. **B. Difference in hit rates between all events and unreferenced events.** To highlight the effect of characters' references to past and future events on participants' retrodictions and predictions, here we display the difference in across-segment mean hit rates between all events and unreferenced events, as a function of temporal distance (lag) to the just-watched segment. **C. Hit rates for unreferenced events.** Participants' (n=37) average response hit rates for unreferenced events are displayed as a function of temporal distance to the just-watched segment. Error bars denote bootstrapped 95% confidence intervals. Panels A–C: colors are described in the Figure 4 caption. **D. Hit rates and counts of referenced and unreferenced events.** As a function of temporal distance to the just-watched segment, the sub-panels display the across-segment mean numbers (x-axes) and hit rates (y-axes) of referenced (red) and unreferenced (gray) events that participants hit (darker shading) or missed (lighter shading) in their uncued retrodictions (top sub-panel) and uncued predictions (bottom sub-panel). Intuitively, the widths of the rectangles at each lag denote the total number of events at each possible lag. The darker shading denotes the proportions of events that participants retrodicted or predicted, and the lighter shading denotes the proportions of events that participants "missed" in their responses.

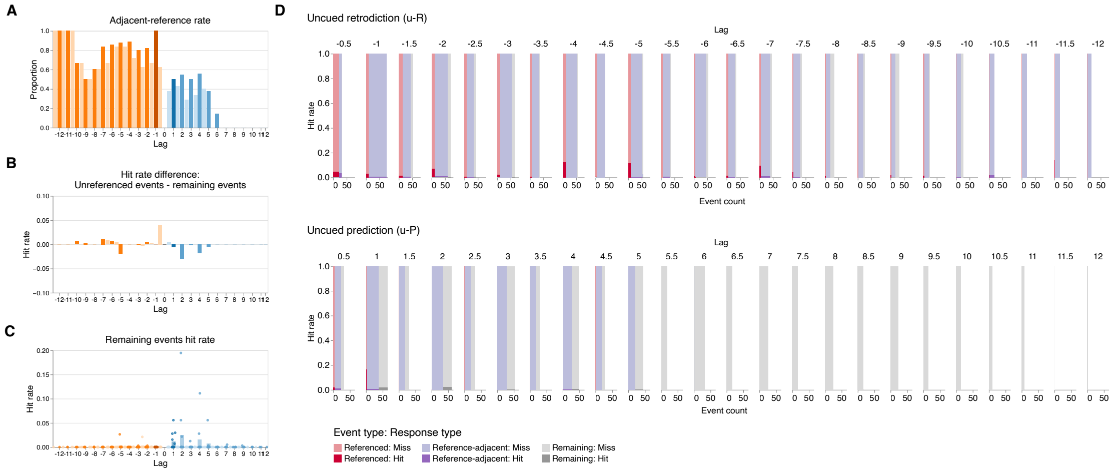

Figure S9: **Hit rates of reference-adjacenet events (replication experiment). A. Adjacent reference rate for unreferenced events as a function of lag.** Across all possible just-watched segments (lag 0), the bar heights denote the average proportion of unreferenced events in other past or future segments that were temporally adjacent to any referenced event. **B. Difference in hit rates between unreferenced events and remaining events.** To highlight the effect of reference adjacency on retrodiction and prediction of unreferenced events, here we display the difference in across-segment mean hit rates between unreferenced events and remaining events, as a function of temporal distance (lag) to the just-watched segment. **C. Hit rates for remaining events.** Participants' (n=37) across-segment mean response hit rates for unreferenced events that were *not* temporally adjacent to any referenced events are displayed as a function of temporal distance to the just-watched segment. Error bars denote bootstrapped 95% confidence intervals. Panels A–C: colors are described in the Figure 4 caption. **D. Hit rates and counts of referenced, reference-adjacent, and remaining events.** As a function of temporal distance to the just-watched segment, the sub-panels display the numbers (x-axes) and proportions (y-axes) of referenced (red), reference-adjacent (purple), and remaining (gray) events that participants hit (darker shading) or missed (lighter shading) in their uncued retrodictions (top sub-panel) and uncued predictions (bottom sub-panel).

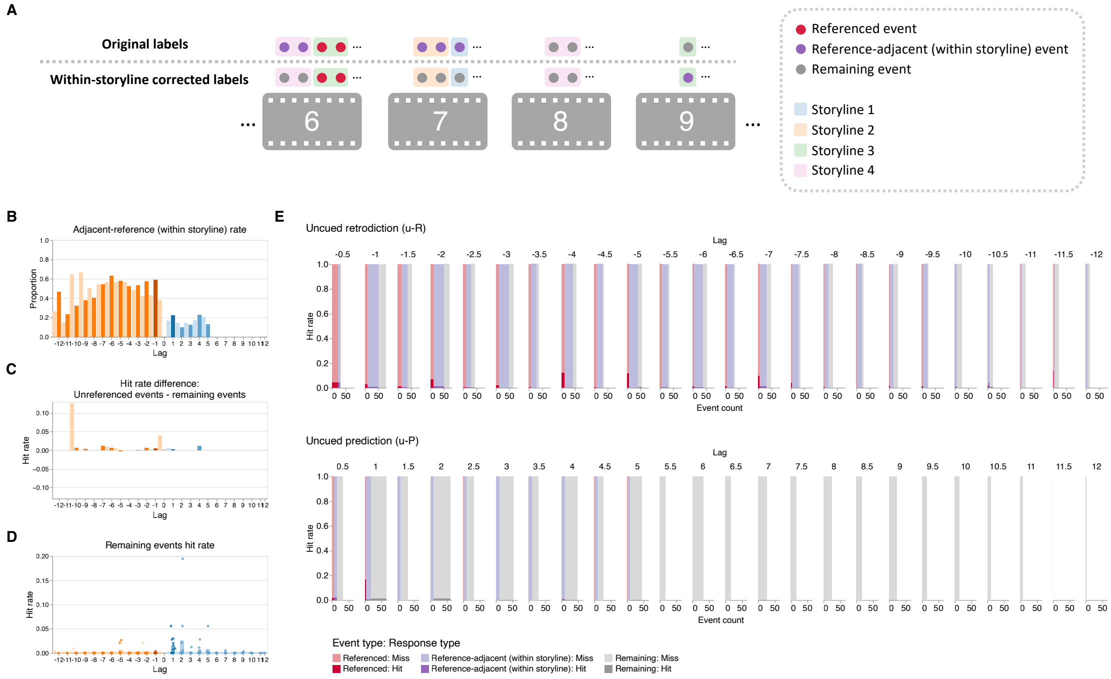

Figure S10: **Within-storyline reference-adjacent events are associated with higher hit rates (replication experiment). A. Illustration of annotation approach.** Rather than labeling reference-adjacent events as whether they were temporally adjacent to a referenced event using the original segment sequences, here we used within-storyline corrected reference-adjacency labels. We determined four storylines in the segments used in the replication experiment and assigned a storyline label for each event (shown as different background colors of each event). We then labeled reference-adjacent events as whether they were temporally adjacent to a referenced event within the same storyline (same background colors). **B. Adjacent reference rate for unreferenced events as a function of lag.** Across all possible just-watched segments (lag 0), the bar heights denote the average proportion of unreferenced events in other past or future segments that were temporally adjacent to any referenced event. **C. Difference in hit rates between unreferenced events and remaining events.** To highlight the effect of reference adjacency on retrodiction and prediction of unreferenced events, here we display the difference in across-segment mean hit rates between unreferenced events and remaining events, as a function of temporal distance (lag) to the just-watched segment. **D. Hit rates for remaining events.** Participants' (n=37) across-segment mean response hit rates for unreferenced events that were *not* temporally adjacent to any referenced events are displayed as a function of temporal distance to the just-watched segment. Error bars denote bootstrapped 95% confidence intervals. Panels A–C: colors are described in the Figure 4 caption. **E. Hit rates and counts of referenced, reference-adjacent, and remaining events.** As a function of temporal distance to the just-watched segment, the sub-panels display the numbers (x-axes) and proportions (y-axes) of referenced (red), reference-adjacent (purple), and remaining (gray) events that participants hit (darker shading) or missed (lighter shading) in their uncued retrodictions (top sub-panel) and uncued predictions (bottom sub-panel).

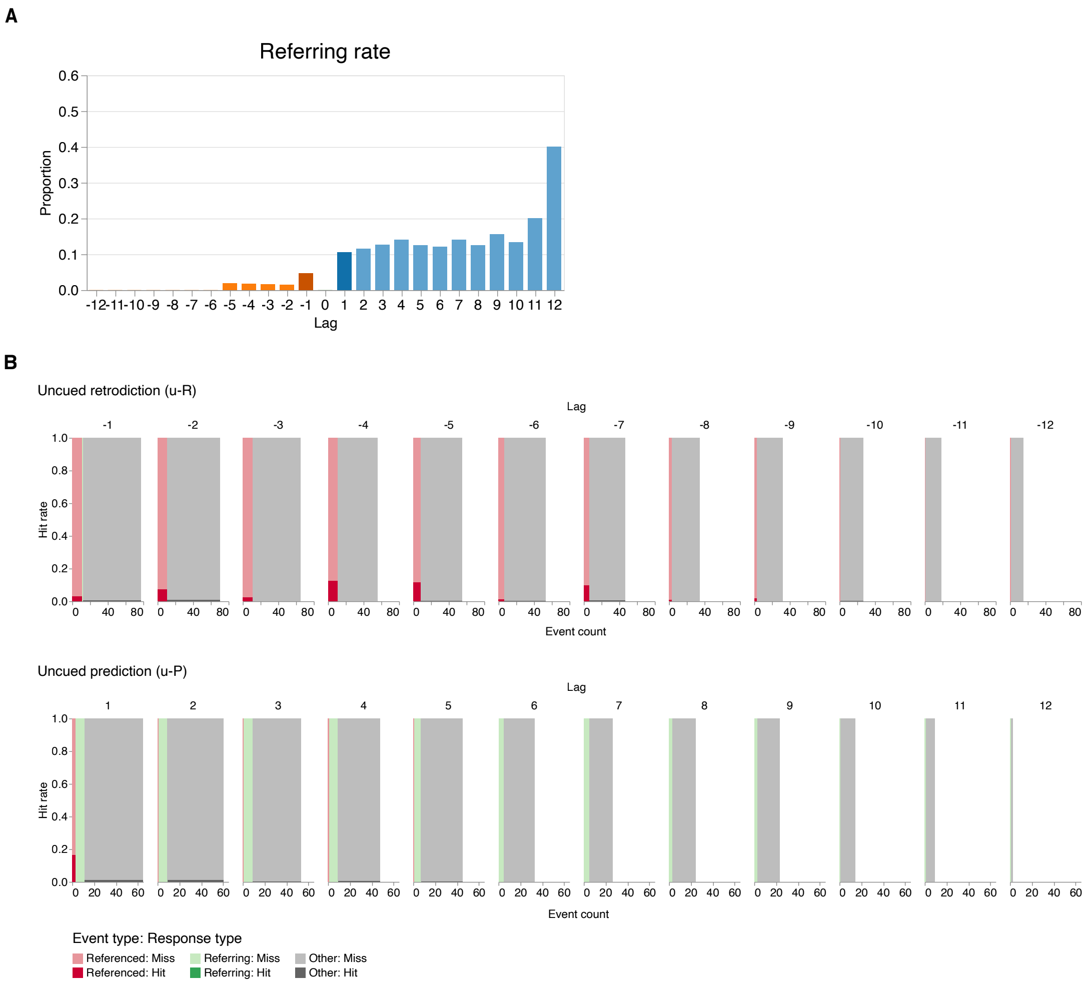

Figure S11: **Hit rates for referenced versus referring events (replication experiment). A. Referring rate as a function of lag.** Across all possible just-watched segments (lag 0), the bar heights denote the across-segment mean proportions of events containing references to events in other past or future segments in our replication experiment's stimuli. The bar colors are described in the Figure 4 caption. **B. Hit rates and counts of referenced, referring, and other events.** As a function of temporal distance to the just-watched segment, the sub-panels display the numbers (x-axes) and hit rates (y-axes) of referenced (red), referring (green), and other (gray) events that participants hit (darker shading) or missed (lighter shading) in their uncued retrodictions (top sub-panel) and uncued predictions (bottom sub-panel).

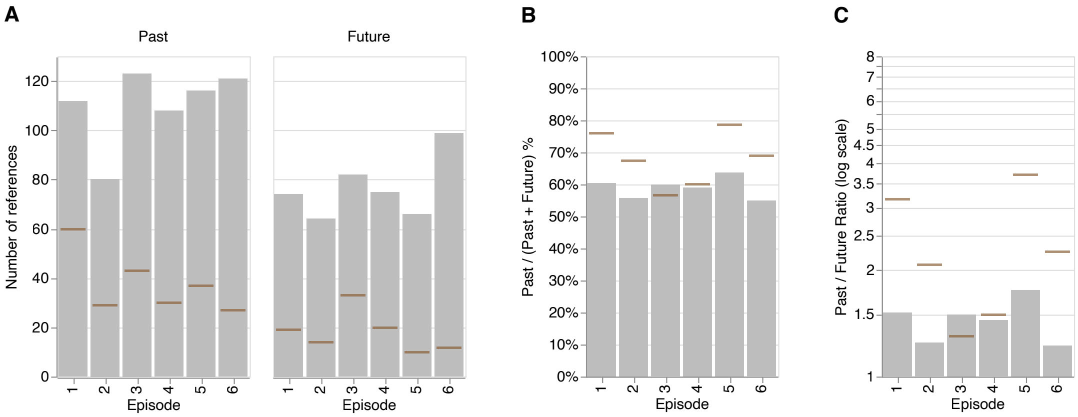

Figure S12: **Comparison of automatic and manual approach for identifying references to past and future events in *The Chair*, Season 1, Episodes 1–6. A. Numbers of references to past and future events.** Ticks indicate manually identified numbers, and bar heights indicate automatically identified numbers. We used Episode 1 from this series as the stimulus in our replication experiment. **B. Proportions of references to past and future events.** Ticks indicate the proportions of references to past events in all references (past + future) based on manually identified numbers, and bar heights indicate the proportions based on automatically identified numbers. **C. Ratios of references to past and future events.** Ticks indicate the ratios of references to past events and future events based on manually identified numbers, and bar heights indicate the ratios based on automatically identified numbers.
