# Supplementary material for: Temporal asymmetries in inferring unobserved past and future events
Source: Nat Commun. 2024 Oct 1;15:8502. doi: 10.1038/s41467-024-52627-5 (PMC11445511; doi:10.1038/s41467-024-52627-5)
Supplement: Supplementary file 3 — Reporting Summary [file 41467_2024_52627_MOESM3_ESM.pdf]

nature portfolio

Corresponding author(s): Jeremy R. Manning

Last updated by author(s): Jun 25, 2024

Reporting Summary

Nature Portfolio wishes to improve the reproducibility of the work that we publish. This form provides structure for consistency and transparency in reporting. For further information on Nature Portfolio policies, see our Editorial Policies and the Editorial Policy Checklist.

Statistics

For all statistical analyses, confirm that the following items are present in the figure legend, table legend, main text, or Methods section.

n/a	Confirmed	
☐	☒	The exact sample size (n) for each experimental group/condition, given as a discrete number and unit of measurement
☐	☒	A statement on whether measurements were taken from distinct samples or whether the same sample was measured repeatedly
☐	☒	The statistical test(s) used AND whether they are one- or two-sided<br>*Only common tests should be described solely by name; describe more complex techniques in the Methods section.*
☐	☒	A description of all covariates tested
☐	☒	A description of any assumptions or corrections, such as tests of normality and adjustment for multiple comparisons
☐	☒	A full description of the statistical parameters including central tendency (e.g. means) or other basic estimates (e.g. regression coefficient) AND variation (e.g. standard deviation) or associated estimates of uncertainty (e.g. confidence intervals)
☐	☒	For null hypothesis testing, the test statistic (e.g. F, t, r) with confidence intervals, effect sizes, degrees of freedom and P value noted<br>*Give P values as exact values whenever suitable.*
☒	☐	For Bayesian analysis, information on the choice of priors and Markov chain Monte Carlo settings
☐	☒	For hierarchical and complex designs, identification of the appropriate level for tests and full reporting of outcomes
☐	☒	Estimates of effect sizes (e.g. Cohen's d, Pearson's r), indicating how they were calculated

Our web collection on statistics for biologists contains articles on many of the points above.

Software and code

Policy information about availability of computer code

Data collection	The main experiment was implemented using jsPsych (v6.1.0) and JATOS (v3.4.1). The replication experiment was implemented using Qualtrics.
Data analysis	All code used to analyze data and generate figures can be found here: https://github.com/ContextLab/prediction-retrodiction-paper. Data were analyzed and visualized in Python using numpy, pandas, scipy, scikit-learn, and Altair. Sentence embeddings were extracted using Google Universal Sentence Encoder (Transformer version v5, https://tfhub.dev/google/universal-sentence-encoder-large/5). Statistical tests were implemented in R using afex and emmeans.

For manuscripts utilizing custom algorithms or software that are central to the research but not yet described in published literature, software must be made available to editors and reviewers. We strongly encourage code deposition in a community repository (e.g. GitHub). See the Nature Portfolio guidelines for submitting code & software for further information.

Data

Policy information about availability of data

All manuscripts must include a data availability statement. This statement should provide the following information, where applicable:

- Accession codes, unique identifiers, or web links for publicly available datasets
- A description of any restrictions on data availability
- For clinical datasets or third party data, please ensure that the statement adheres to our policy

All data in the study can be found here: https://github.com/ContextLab/prediction-retrodiction-paper.

Research involving human participants, their data, or biological material

Policy information about studies with human participants or human data. See also policy information about sex, gender (identity/presentation), and sexual orientation and race, ethnicity and racism.

Reporting on sex and gender	Self-report gender data were collected. A total of 36 participants (25 female, 11 male) were recruited in the main experiment. A total of 37 participants (21 female, 16 male) were recruited in the main experiment. We had no a priori hypotheses about gender differences in this study, thus we did not conduct any gender-based analysis.
Reporting on race, ethnicity, or other socially relevant groupings	We didn't use socially constructed or socially relevant categorization variables in the manuscript. The main experiment used a within-participant and in the replication experiment we randomly assigned participants into conditions.
Population characteristics	See below.
Recruitment	Participants were recruited from the Dartmouth College community using Sona. Participants received course credit or monetary compensation for their time. We are not aware of any biases in our recruiting protocols.
Ethics oversight	All of our protocols were approved by the Committee for the Protection of Human Subjects at Dartmouth College

Note that full information on the approval of the study protocol must also be provided in the manuscript.

Field-specific reporting

Please select the one below that is the best fit for your research. If you are not sure, read the appropriate sections before making your selection.

☐ Life sciences ☒ Behavioural & social sciences ☐ Ecological, evolutionary & environmental sciences

For a reference copy of the document with all sections, see nature.com/documents/nr-reporting-summary-flat.pdf

Behavioural & social sciences study design

All studies must disclose on these points even when the disclosure is negative.

Study description	Mixed-methods study where text data were collected and analyzed with both quantitative and qualitative methods.
Research sample	Participants (main experiment: 25 female, mean age 21.47 years, range 19–50 years; replication experiment: 21 female, mean age 22.24 years, range 19–30) were recruited from the Dartmouth College community. Participants have various ethnic backgrounds. This study sample was chosen for convenience (self registration via Sona).
Sampling strategy	The study used a convenience sample. Participants signed up via Sona and received course credit or monetary compensation for their time. Due to the novelty of the experimental design, the sample size was not predetermined. We aimed to strike a balance between the sample sizes of both participants and stimuli, and we modeled participants and stimuli as crossed random effects in (generalized) linear mixed models. We also conducted a replication experiment.
Data collection	The experiment was conducted in a sound- and light-attenuated testing room. Videos were displayed using a 27-inch iMac desktop computer (resolution: 5120 × 2880) and sound was presented using the iMac's built-in speakers. Data were collected using JATOS (main experiment) and Qualtrics (replication experiment). The experimenter was present during the training session (main experiment), but was absent during the experimental session and was blind to the experimental conditions.
Timing	Data for the main experiment were collected between Jan, 2020 and Feb, 2020. Data for the replication experiment were collected between May, 2023 and July, 2023.
Data exclusions	We discarded a small number (main experiment: n = 20; replication experiment: n = 6) of responses in the character-cued condition that did not contain references to all cued characters, along with one additional response due to the participant's misunderstanding of the task instructions during that trial.
Non-participation	One participant dropped out due to deciding that they preferred not to watch the TV episodes we showed in our experiment.

Randomization	Participants were randomly assigned to experimental conditions.

Reporting for specific materials, systems and methods

We require information from authors about some types of materials, experimental systems and methods used in many studies. Here, indicate whether each material, system or method listed is relevant to your study. If you are not sure if a list item applies to your research, read the appropriate section before selecting a response.

Materials & experimental systems

n/a	Involved in the study
☒	☐ Antibodies
☒	☐ Eukaryotic cell lines
☒	☐ Palaeontology and archaeology
☒	☐ Animals and other organisms
☒	☐ Clinical data
☒	☐ Dual use research of concern
☒	☐ Plants

Methods

n/a	Involved in the study
☒	☐ ChIP-seq
☒	☐ Flow cytometry
☒	☐ MRI-based neuroimaging

Plants

Seed stocks	*Report on the source of all seed stocks or other plant material used. If applicable, state the seed stock centre and catalogue number. If plant specimens were collected from the field, describe the collection location, date and sampling procedures.*
Novel plant genotypes	*Describe the methods by which all novel plant genotypes were produced. This includes those generated by transgenic approaches, gene editing, chemical/radiation-based mutagenesis and hybridization. For transgenic lines, describe the transformation method, the number of independent lines analyzed and the generation upon which experiments were performed. For gene-edited lines, describe the editor used, the endogenous sequence targeted for editing, the targeting guide RNA sequence (if applicable) and how the editor was applied.*
Authentication	*Describe any authentication procedures for each seed stock used or novel genotype generated. Describe any experiments used to assess the effect of a mutation and, where applicable, how potential secondary effects (e.g. second site T-DNA insertions, mosiacism, off-target gene editing) were examined.*
